# PinX1 regulation of telomerase activity and apoptosis in nasopharyngeal carcinoma cells

**DOI:** 10.1186/1756-9966-31-12

**Published:** 2012-02-08

**Authors:** Xiao-Fen Lai, Cong-Xiang Shen, Zhong Wen, Yu-Hong Qian, Chao-Sheng Yu, Jun-Qi Wang, Ping-Neng Zhong, Hai-Li Wang

**Affiliations:** 1 Department of Otolaryngology Head and Neck Surgery, Zhujiang Hospital, Southern Medical University, Guangzhou 510282, China; 2 Department of Otolaryngology Head and Neck Surgery, Zhujiang Hospital, Southern Medical University, 253# GongYe Road, Guangzhou 510282, China

**Keywords:** PinX1, Telomerase inhibitor, Proliferation, Migration, Apoptosis, Cell cycle, Human nasopharyngeal carcinoma

## Abstract

**Background:**

Human interacting protein X1 (PinX1) has been identified as a critical telomerase inhibitor and proposed to be a putative tumor suppressor gene. Loss of PinX1 has been found in a large variety of malignancies, however, its function in inhibiting telomerase activity of tumor cells is not well documented. Here we show that PinX1 is essential for down-regulation telomerase activity of nasopharyngeal carcinoma.

**Methods:**

Expression vectors of human PinX1 (pEGFP-C3-PinX1) and its small interfering RNA (PinX1-FAM-siRNA) were constructed and transfected into NPC. Their effects on mRNA of telomerase catalytic subunit (hTERT), telomerase activity, cell proliferation, cell migration, wound healing, cell cycles and apoptosis were examined using semi-quantitative RT-PCR, stretch PCR, MTT assay, Transwell, scratch assay and flow cytometry, respectively.

**Results:**

Transfection of pEGFP-C3-PinX1 and PinX1-FAM-siRNA increased and reduced PinX1 mRNA by 1.6-fold and 70%, respectively. Over-expression of PinX1 decreased hTERT mRNA by 21%, reduced telomerase activity, inhibited cell growth, migration and wound healing ability, arrested cells in G0/G1 phase, and increased apoptotic index. In contrast, down-regulation of PinX1 did not alter the above characteristics.

**Conclusions:**

PinX1 may play important roles in NPC proliferation, migration and apoptosis and has application potential in tumor-targeted gene therapy.

## Background

Nasopharyngeal carcinoma (NPC) is one of the most incident and dangerous malignant tumors in southern provinces of China. Genetic factors and environmental factors including Epstein-Barr virus are the two major risk factors for NPC. Radiotherapy along with other auxiliary methods such as chemotherapy is used to treat NPC. Although equipments and technologies in radiotherapy and chemotherapy have been greatly advanced in recent years, the 5-year survival rate of patients with NPC remains about 70%. In addition, systemic and local side effects caused by chemotherapy greatly humbled the patient physically and psychologically. Therefore, it is of importance to study the etiology of NPC and explore new, safe and effective modalities for NPC therapy.

Telomerase is well known for its role in the development of malignant tumors. Studies from our group and others [1,2] have found enhanced mRNA level of telomerase catalytic subunit (TERT) and telomerase expression in 88% of NPC specimens and NPC cell line HNE1.

Antisense nucleic acid targeted to telomerase RNA and hTERT can inhibit telomerase activity and induce apoptosis of tumor cells [3,4].

Suicide genes TK and CD are powerful in cancer gene therapy. However, their application has been limited due to lack of targeting. Using targeted promoter such as hTERT promoter to regulate suicide gene expression has been a direction in tumor gene therapy. In recent years, we have constructed tumor specific TK expression and enhanced expression vectors using hTERT promoter and found that transfection of these vectors could specifically kill NPC and its stem cells *in vitro *and inhibit NPC exograft in null mice in vivo without damaging normal cells and mouse liver and kidney [5-7], indicating that inhibition of telomerase activity is a key step to in NPC treatment.

Study on telomerase inhibitors has become an important area in targeted tumor gene therapy. Pin2/TRF1 interacting protein X1 (PinX1) was recently found as a tumor suppressor and telomerase inhibitor *in vivo*. It is expressed in normal human tissues, but not or less expressed in tumor tissues. Studies have found that PinX1 can inhibit telomerase activity in gastric and liver tumor cells and induce their apoptosis [8-11]. The expression of PinX1 has been positively correlated with telomerase activity in leukemia [12,13]. However, some studies on prostate cancer, gastrointestinal cancer and medulloblastoma indicate that gene polymorphism rather than PinX1 expression is the key factor in inhibiting telomerase [14-16] and PinX1 as a microtubule binding protein plays an important role in stabilizing chromosome [17]. In short, the mechanisms by which PinX1 regulates telomerase/telomere in tumor cells are complex and may vary in different tumors.

The effect of PinX1 on NPC apoptosis and the mechanisms by which PinX1 affects telomerase activity have not been reported. Therefore, in this study, we constructed PinX1 expression vector and utilized its small interfering RNA to study its possible role in NPC.

## Methods

### Materials

Austria newborn calf serum, RT-PCR kit and DNA marker were from Takara Biotechnology Co., Ltd. Tetrazolium blue (MTT) was from Sigma. Lipofectamine 2000™ and RNA extraction reagent Trizol were from Invitrogen (USA). Transwell cell culture plates were from Corning (USA). Plasmid extraction kit was from Tiangen Biotech (Beijing) Co. Ltd. Telomerase activity detection kit was from Toyobo Corporation.

### Cell lines

Human nasopharyngeal carcinoma 5-8 F cells (NPC 5-8 F) and human vascular endothelial cells (VEC) were maintained in RPMI 1640 and DMEM, respectively, supplemented with 10% calf serum, 100 U/mL penicillin and 100 U/mL streptomycin at 37°C in a 5% CO_2 _incubator as previously reported. After passaged using conventional method, cells were used for experiment at logarithmic phase.

### Plasmid construction

Synthesized PinX1 DNA was inserted into pEGFP-C3 vector at XhoI and EcoRI sites. Recombinant plasmid was transformed into *E. coli *DH5α and screened by kanamycin and neomycin resistance. Possitive pEGFP-C3-PinX1 was further identified by XhoI and EcoRI digestion and confimed by DNA sequencing.

### PinX1 siRNA

PinX1 siRNAs were designed using online software from Invitrogen company (http://maidesigner.Invitrogen.com/maiexpress/). After blast and analysis for homology in human genome, three siRNAs PinX1-963, PinX1-695 and PinX1-242 were selected and used to silence PinX1. Preliminary experiments indicated that PinX1-695 with sense sequence of 5'-GUAAAGAUGUGGAAAGUUATT-3' and anti-sense sequence of 5'-TTCAUUUCUACACCUUUCAAU-3' could effectively downregulate PinX1. Threrefore, it was synthesized as FAM-labeled siRNA and used in all experiments.

### Experimental design and cell transfection

Cells at logarithmic phase were innoculated into 6-well plated cultured in media without antibiotics for 24 h to reach 80-90% confluency. Cells were then transfected with pEGFP-C3-PinX1, and PinX1-FAM-siRNA using lipofectimaine 2000™ according to the protocol provided by the manufacturer. Untransfected cells and cells treated with lipofectimine 2000™ alone and cells transfected with pEGFP-C3 were used as controls. Cells were observed 24-48 h after transfection under fluorescence microscope to examine transfection efficiency.

### RNA isolation and measurement of PinX1 and hTERT mRNA levels by RT-PCR

Total RNA was extracted with Trizol 48 h after transfection following the manufacturer's instruction. Four μL mRNA of each sample was reverse transcribed into cDNA by AMV reverse transcriptase and used as template in RT-PCR. PCR condition used for PinX1 and internal reference GAPDH was 94°C for 2 min followed by 25 cycles of 94°C for 1 min, 55°C for 1 min and 72°C for 2 min, and 72°C for 5 min. PCR condition used for hTERT and its internal reference GAPDH was 94°C for 4 min followed by 30 cycles of 94°C for 30 s, 49°C for 30 s and 72°C for 45 s and 72°C for 5 min. The specific primers used in these reactions were followings: PinX1 forward 5' TTTTCTCGAGATGTCTATGCTGGCTGAACG 3' and reverse 5' TTTTGAATTCTCATTTGGAATCTTTCTTC 3'; hTERT forward 5' CCGAGTGACCGTGGTTTCTGTG 3' and reverse 5'GGAAGCGGCGTTCGTTGTG 3' and GAPDH forward 5' GGAAGATGGTGATGGGATT 3' and reverse 5' GGATTTGGTCGTATTGGG 3'. The expected PCR products were 987 bp, 670 bp and 205 bp for PinX1, hTERT and GAPDH, respectively. The amplicons were analyzed by electrophoresis, imaged using UVI gel imaging system and quantified using Quantity one software. Expression levels of PinX1 and hTERT were normalized to internal reference GAPDH.

### Measurement of cell proliferation by MTT

NPC 5-8 F cells at logarithmic phase were inoculated into 96-well plate with 1 × 10^5 ^cells in each well. Cell viability at 0 h, 24 h, 48 h and 72 h was examined using MTT method. OD_490nm _values at each time point were detected in six duplicate wells and their averages were used to plot growth curve and calculate the growth inhibition rate of each treatment using the following formula:

$$\begin{array}{c} \text{Growth inhibition rate Ir = O}{\text{D}}_{\text{490nm}}\text{ of the control group }-\text{ O}{\text{D}}_{\text{490nm}}\text{ of the treatment group}\\ \;\;\;\;\;\;\;\;\;\;/\text{O}{\text{D}}_{\text{490nm}}\text{ of the control group }\times \text{ 100\% } \end{array}$$

### Detection of telomerase activity by stretch PCR

NPC 5-8 F cells at logarithmic phase were inoculated into 6-well plate with 1 × 10^6^/well. 24 h later, cells were transfected as described above. 48 h after transfection, telomerase activity was measured using stretch PCR assay based on the protocol provided by the manufacturer. Meanwhile, telomerase activity in control ECV-304 cells was similarly examined.

### Effect of PinX1 on cell migration

Cell migration was examined using transwell. In detail, NPC 5-8 F cells at logarithmic phase were starved overnight in serum free RPMI 1640 media. Cells were deattached with 0.25% trypsin. After wash with PBS, they were resuspended in RPMI 1640 containing 1 mmol/L CaCl_2_, 1 mmol/L MgCl_2_, 0.2 mmol/L MnCl_2 _and 5 g/L BSA, and adjusted to 1 × 10^5^/mL. 200 μL cell suspension was added into the upper chamber of the transwell and 500 μL RPMI 1640 containing 10% newborn calf serum (as a chemokine) was added into the lower chamber of the transwell. The transwell was then cultured at 37°C in a incubator supplemented with 5% CO_2_. 24 h later, cells on the upper surface of polycarbonate membrane of the transwell were removed with a cotton swab and the cells that migrated onto the lower surface of the membrane were fixed with 4% paraformaldehyde for 15 min, washed three times with PBS for 5 min each and stained with crystallization violet for 3 min. After further wash with PBS, the membrane was air dried and cell number on the membrane was counted under microscope at 400 magnification. The number of migrated cells was expressed as the average of five randomly selected fields.

### Scratch assay

Transfected NPC 5-8 F cells at logarithmic phase were inoculated in 6-well plate pre-coated with collagen IV. When monolayer was formed, cells were scratched with a 100 μL tip and cultured in media containing 10% FBS. Zero, 12, 24, and 36 h after scratching, cells in each well were photographed under microscope. The distances between the two edges of the scratched cells in four fields were measured and the average distance was used to calculate the healing rate using the following formula:

$$\begin{array}{c} \text{Healing rate = the distance befor healing }-\text{the distance after healing}\\ \;\;\;\;\;\;\text{/the distance before healing)}\times \text{100\% }\text{.} \end{array}$$

### Measurement of cell cycle and apoptosis by flow cytometry

48 h after transfection, NPC 5-8 F cells were collected, washed with PBS, resuspended in PBS at 1 × 10^6^/mL, and stained with Annexin V and propidium iodide solution (PI) for 15 min at dark. Apoptotic cells were then analyzed by flow cytometry and apoptotic index (AI) was calculated using AI = apoptotic cells/total cells × 100%. Cell cycle was determined after fixing with pre-cooled 75% ethanol at 4°C and wash with PBS.

### Statistical analysis

Data were expressed as mean ± standard variation $\bar{\text{X}}\pm \text{s}$ and analyzed using SPSS13.0 statistical software package. Differences between samples in RT-PCR, telomerase activity, migration assay, scratch assay, cell cycle and apoptosis assay were tested using single factor analysis of variance and LSD method for multiple comparisons. Differences in between samples in proliferation assay or scratch assay were tested using factorial design analysis of variance and Dunnett's T3 method for multiple comparisons. A *p *value less than 0.05 was considered as significant difference. Before comparison, data homogeneity of variance was first examined using F test. In the case of heterogeneity of variance, the approximate variance F test/Welch method was used.

## Results

**Figure 1:**
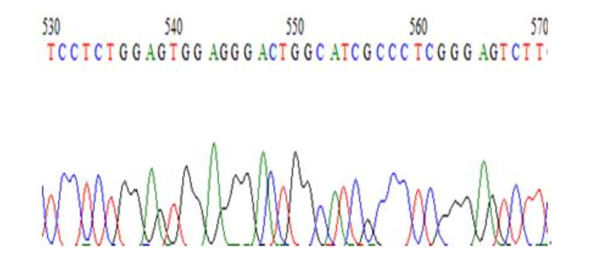
**The sequencing map of PinX1 gene**.

We first confirmed the successful construction of PinX1 expression vector pEGFP-C3-PinX1 by digestion with both XhoI and EcoRI and bi-directional sequence analysis, As shown in Figure 1.

**Figure 2:**
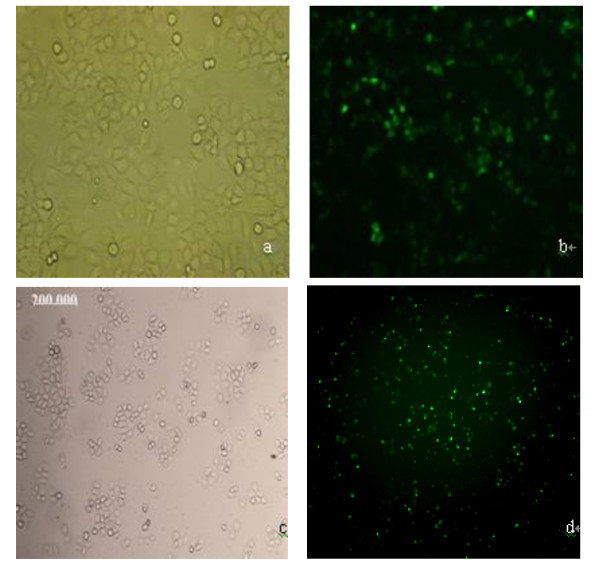
**Images of nasopharyngeal carcinoma 5-8 F cells transfected with plasmid pEGFP-C3-PinX1 under bright field (a) and fluorescent field (b) and transfected with PinX1-FAM-siRNA under bright field (c) and fluorescent field (d)**.

We then examined the transfection efficient under fluorescence microscope. As shown in Figure 2, above 50% of cells were transiently transfected.

**Figure 3:**
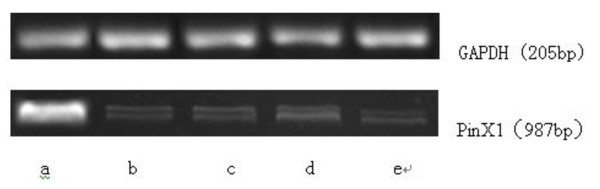
**Electrophoresis analysis of RT-PCR amplicons from PinX1 mRNA isolated from nasopharyngeal carcinoma 5-8 F cells transfected with (a) pEGFP-C3-PinX1, (b) pEGFP-C3, (c) PinX1-FAM-siRNA, respectively, and treated with (d) lipofectamine alone and (e) control, respectively, showing relative PinX1 mRNA level**.

We next detected PinX1 mRNA level in tranfected cells by RT-PCR. As shown in Figure 3, an expected fragment of 987 bp was amplified in samples isolated from non-transfected NPC 5-8 F cells, lipofectamine treated cells, and cells transfected with pEGFP-C3-PinX1 and pEGFP-C3, respectively, but not in NPC 5-8 F cells transfected with PinX1-FAM-siRNA. Its intensity was the strongest in cells transfected with pEGFP-C3-PinX1. As shown in Table 1, PinX1 mRNA level in cells transfected with pEGFP-C3-PinX1 is 1.6-fold of that in untreated cells (*p *< 0.05). By contrast, PinX1 mRNA level in cells transfected with PinX1-FAM-siRNA reduced by 70% compared with that in untreated cells (*p *< 0.05). In addition, PinX1 mRNA level in cells treated with lipofectimine alone or transfected with pEGFP-C3 was not significantly changed (*p *> 0.05).

**Table 1:** PinX1 mRNA levels $(\bar{\text{X}}\pm \text{s)}$

Sample	mRNA	F	*P*
pEGFP-C3-PinX1	1.601 ± 0.166*	24.756	0.00
pEGFP-C3	1.223 ± 0.148		
Lipofectamine alone	1.042 ± 0.166		
Untreated	1.000 ± 0.000		
PinX1-FAM-siRNA	0.304 ± 0.055**		

* vs untreated, *P *< 0.001; ** vs untreated, *P *< 0.05. PinX1 mRNA level was normalized to GAPDH.

**Figure 4:**
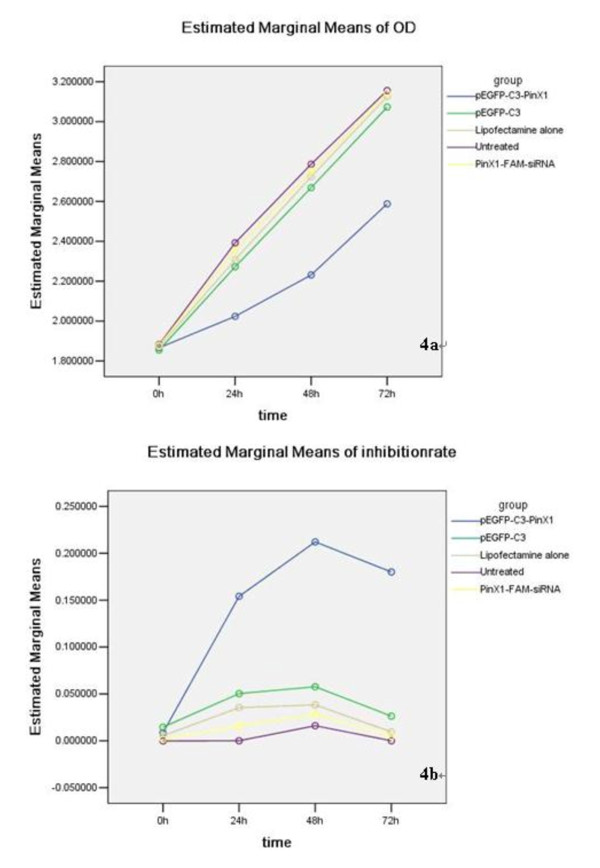
**Growth curves of nasopharyngeal carcinoma 5-8 F cells transfected with pEGFP-C3-PinX1, pEGFP-C3, PinX1-FAM-siRNA and treated with lipofectamine alone, indicating that PinX1 overexpression significantly inhibited NPC 5-8 F cell growth**.

Having confirmed that transfection of pEGFP-C3-PinX1 and PinX1-FAM-siRNA could significantly enhance and reduce PinX1 mRNA, respectively, we then explored their effects on NPC 5-8 F cell proliferation using MTT assay. As shown in Table 2, factorial design analysis of variance found that the mean value of OD_490nm _in cells transfected with pEGFP-C3-PinX1 was 2.15, which was significantly decreased compared with that of 2.52 and 2.50 in untreated NPC 5-8 F cells and cells transfected with PinX1-FAM-siRNA, respectively (F = 31.504, *p *= 0.000). In addition, there was no significant difference in the mean value of OD_490nm _in untreated cells, lipofectimine treated cells, cells transfected with pEGFP-C3 and cells transfected with PinX1-FAM-siRNA. Figure 4 shows the cell growth curve by plotting the OD_490nm _value *vs *time (0 h, 24 h, 48 h and 72 h). Cell growth rate calculated based on the curve indicated that overexpression of PinX1 significantly inhibited the growth of NPC 5-8 F cells, whereas downregulation of PinX1 by siRNA transfection did not affect the growth of NPC 5-8 F cells.

**Table 2:** OD_490nm _value of NPC 5-8 F cells $(\bar{\text{X}}\pm \text{s)}$

Sample	Time				Total	Welch/F Value	*P *Value
				
	0 h	24 h	48 h	72 h			
pEGFP-C3-PinX1	1.86 ± 0.07	2.02 ± 0.11	2.23 ± 0.08	2.58 ± 0.03	2.15 ± 0.27	74.246	0.000
pEGFP-C3	1.85 ± 0.04	2.27 ± 0.17	2.66 ± 0.15	3.07 ± 0.23	2.44 ± 0.47	57.327	0.000
Lipofectamine alone	1.87 ± 0.05	2.30 ± 0.10	2.72 ± 0.13	3.12 ± 0.08	2.48 ± 0.47	156.436	0.000
Untreated	1.88 ± 0.02	2.39 ± 0.23	2.78 ± 0.19	3.15 ± 0.12	2.52 ± 0.50	189.669Δ	0.000
PinX1-FAM-siRNA	1.87 ± 0.01	2.35 ± 0.05	2.75 ± 0.04	3.14 ± 0.12	2.50 ± 0.47	720.110Δ	0.000
Total	1.87 ± 0.04	2.27 ± 0.19	2.63 ± 0.24	3.01 ± 0.25	2.42 ± 0.46	437.621*	0.000
Welch/F value	0.309	5.696	35.155Δ	5.600	35.870*	F = 4.592#	
P value	0.869	0.002	0.000	0.000	0.000	*P *= 0.000#	

*F and *P *values of major effect; # F and *P *values of interaction effects; Δ: F-test based on heterogeneity of variance. Note: OD value is propotinal to the number of live cells and indirectly reflects cell proliferation.

**Figure 5:**
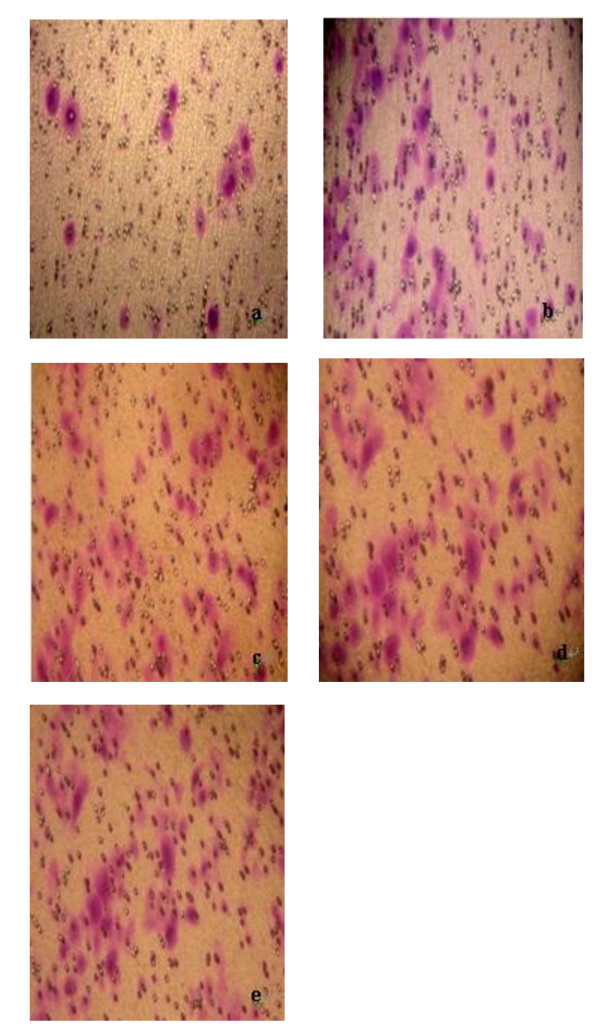
**Effect of PinX1 on nasopharyngeal carcinoma cell migration**. Data were presented as mean number of cells migrated onto the lower surface of transwell counted in five randomly selected fields under microscope. a: NPC 5-8 F cells transfected with pEGFP-C3-PinX1; b: NPC 5-8 F cells transfected with pEGFP-C3; c: NPC 5-8 F cells treated with lipofectamine alone; d: untreated NPC 5-8 F cells; e: NPC 5-8 F cells transfected with PinX1-FAM-siRNA.

We further explored the effect of PinX1 on NPC 5-8 F cell migration. As shown in Table 3 and Figure 5, overexpression of PinX1 by transfecting pEGFP-C3-PinX1 significantly decreased NPC 5-8 F migration compared with untreated cells (F = 17.162, *p *= 0.000). By contrast, attenuated Pin X1 expression by transfection of PinX1-FAM-siRNA did not affect NPC 5-8 F cell migration (*p *> 0.05). In addition, transfection of pEGFP-C3 and treatment with lipofectimine alone did not alter the ability of NPC 5-8 F migration (*p *> 0.05).

**Table 3:** Chemotaxic activity of NPC cells in each group $(\bar{\text{X}}\pm \text{s)}$

Sample	Chemotaxic activity (cell number)	F	*P*
pEGFP-C3-PinX1	17.75 ± 5.07*		
pEGFP-C3	30.05 ± 7.22		
Lipofectamine alone	33.90 ± 7.92	17.162	0.000
Untreated	33.20 ± 8.61		
PinX1-FAM-siRNA	33.50 ± 7.60**		

*vs untreated, *P *< 0.001; ** vs untreated, *P *> 0.05.

**Figure 6:**
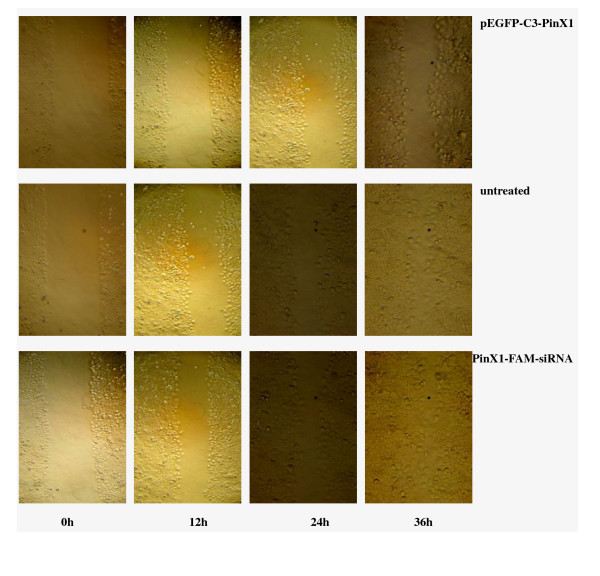
**Effect of PinX1 on wound healing ability of nasopharyngeal carcinoma 5-8 F cells in scratch assay**. Cells transfected with pEGFP-C3-PinX1 (a), pEGFP-C3 (b) and PinX1-FAM-siRNA(e), treated with lipofactamine alone (c), and untreated (d) were inoculated in 6-well plates pre-coated with collagen IV, cultured in media containing 10% newborn calf serum till forming monolayer, then scratched and photographed at 0 h, 12 h, 24 h and 36 h after scratching. The results show that overexpression of PinX1 by transfection of pEGFP-C3-PinX1 significantly increased the wound healing time of NPC 5-8 F cells, while downregulation of PinX1 by transfection of FAM-siRNA reduced has no effect on wound healing.

We next studied the effects of PinX1 on wound healing ability. As shown in Figure 6, factorial design analysis of variance showed that overexpression of PinX1 by transfection of pEGFP-C3-PinX1 significantly attenuated the mean healing rate of untreated 5-8 F cells from 37.80% to 23.74%, and the healing rates at 12 h, 24 h and 36 h (*p *< 0.001). By contrast, the healing rate of NPC 5-8 F cells was not affected by treatment of lipofectamine alone and transfection of pEGFP-C3 and PinX1-FAM-siRNA (*p *> 0.05).

**Figure 7:**
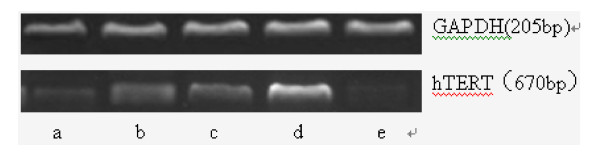
**Effects of PinX1 on hTERT mRNA level in NPC 5-8 F cells**. PinX1 mRNA levels in NPC 5-8 F cells transfected with (a) pEGFP-C3-PinX1, (b) with pEGFP-C3, (c) treated with lipofectamine alone, (d) untreated and (e) transfected with PinX1-FAM-siRNA were measured in RT-PCR and normalized to internal control GAPDH. Data were presented as mean value of three experiments showing that overexpression of PinX1 significantly decreased hTERT mRNA level.

**Figure 8:**
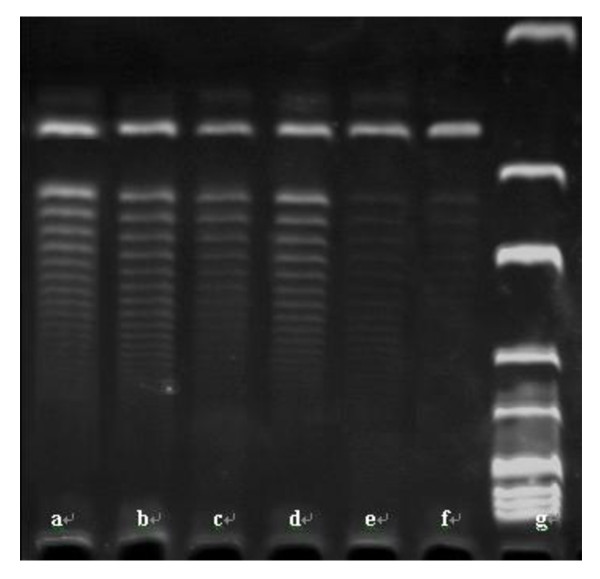
**Effect of PinX1 on telomerase activity in nasopharyngeal carcinoma cells**. Telomerase activity was measured by scratch assay in NPC 5-8 F cells (a) transfected with PinX1-FAM-siRNA, (b) untreated, (c) treated with lipofectamine alone, (d) transfected with pEGFP-C3, (e) transfected with pEGFP-C3-PinX1, and (f) human umbilical vein cells VEC-304. Lane (g) shows the DNA marker. The results indicate that telomerase activity is weak in ECV-304 and strong in untreated NPC 5-8 F cells and overexpression of PinX1 by transfection of pEGFP-C3-PinX1 significantly inhibited telomerase activity in NPC cells, but not affected by transfection of PinX1-FAM-siRNA and pEGFP-C3, and treatment with lipofectamine.

We then examined the effect of PinX1 on hTERT mRNA level and telomerase activity. As shown in Tables 4 and 5 and Figures 7 and 8, overexpression of Pin X1 by transfection of pEGFP-C3-PinX1 significantly reduced hTERT mRNA level by 21% and decreased telomerase activity in NPC 5-8 F cells (*p *= 0.000). By contrast, reduced PinX1 by transfection of PinX1-FAM-siRNA had effects on neither hTERT mRNA level nor telomerase activity in NPC 5-8 F cells (*p *> 0.05). In addition, hTERT mRNA level and telomerase activity in NPC 5-8 F cells were not affected by transfection of pEGFP-C3 and treatment of lipofectamine alone.

**Table 4:** hTERT mRNA level in each group $(\bar{\text{X}}\pm \text{s)}$

Sample	hTERT mRNA	F	*P*
pEGFP-C3-PinX1	0.789 ± 0.024*	117.689	0.000
pEGFP-C3	0.978 ± 0.011		
Lipofectamine alone	0.987 ± 0.014		
Untreated	1.000 ± 0.000		
PinX1-FAM-siRNA	1.001 ± 0.085**		

* vs untreated, *P *< 0.001, ** vs untreated, *P *> 0.05. hTERT mRNA level was normalized to GAPDH.

**Table 5:** Telomerase activity in NPC cells $(\bar{\text{X}}\pm \text{s)}$

Samples	Telomerase activity	F	*P*
pEGFP-C3-PinX1	36227.63 ± 2181.748*	53.816	0.000
pEGFP-C3	58346.993 ± 2181.748		
Lipofectamine alone	59697.199 ± 2181.748		
Untreated	62552.354 ± 2181.748		
PinX1-FAM-siRNA	63600.608 ± 2181.748**		

* vs untreated, *P *< 0.001; ** vs untreated, *P *> 0.05.

We next examined the effect of PinX1 on cell cycle by flow cytometry. As shown in Table 6, overexpression of PinX1 by transfection of pEGFP-C3-PinX1 significantly increased the percentage of NPC 5-8 F cells at G0/G1 phase from 43.0% to 64.0% (*p *< 0.001). However, downregulation of Pin X1 by transfection of PinX1-FAM-siRNA, liopafectamine treatment, and transfection of pEGFP-C3 did not affect the percentage of NC 5-8 F cells at G0/G1 phase.

**Table 6:** Percentage of NPC cells in G0/G1 period $(\bar{\text{X}}\pm \text{s)}$

Samples	NPC in G0/G1 period (%)	F	*P*
pEGFP-C3-PinX1	64.000 ± 3.905*	50.006	0.000
pEGFP-C3	43.900 ± 2.193		
Lipofectamine alone	42.966 ± 1.069		
Untreated	43.033 ± 1625		
PinX1-FAM-siRNA	42.833 ± 1.484**		

* vs untreated, *P *< 0.001; ** vs untreated, *P *> 0.05.

**Figure 9:**
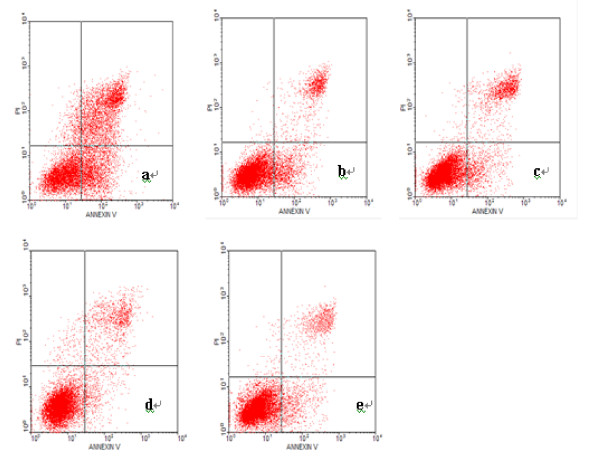
**Effect of PinX1 on nasopharyngeal carcinoma cell apoptosis measured by flow cytometry**. Shown are the diagram of flow cytometry of NPC 5-8 F cells stained with Annexin V and propidium iodide solution (PI) and (a) transfected with pEGFP-C3-PinX1, (b) transfected with pEGFP-C3, (c) treated with lipofectamine alone, (d) untreated and (e) t transfected with PinX1-FAM-siRNA, respectively. The upper and lower right quadrants represent apoptotic cells and the lower left quadrant represents normal cells. The data indicate that the number of apoptotic cells transfected with pEGFP-C3-PinX1 was significantly greater than that of cells treated otherwise.

We last examined the effect of PinX1 on NPC 5-8 F apoptosis by Annexin V/PI staining. Living cells were Annexin V(-)/PI(-) at the lower left quadrant in flow cytometry diagram. Cells with Annexin V(+)/PI(-) at the lower right quadrant were at the early apoptotic status; cells with Annexin V(-)/PI(+) at the upper right quadrant were at late apoptotic status. As shown in Table 7 and Figure 9, overexpression of PinX1 by transfection of pEGFP-C3-PinX1 significantly enhanced AI from 19.266 ± 0.763% in untreated cells and 19.566 ± 0.577% in pEGFP-C3 transfected cells to 49.73 ± 2.ddxzr70% (*p *< 0.01). In addition, there was no difference of AI among untreated cells, cells transfected with pEGFP-C3 and cells treated with lipofectamine (*p *> 0.05).

**Table 7:** Apoptotic index of NPC cells $(\bar{\text{X}}\pm \text{s)}$

Samples	Apoptotic index	F	*P*
pEGFP-C3-PinX1	49.733 ± 2.702*	183.419	0.000
pEGFP-C3	19.566 ± 0.577		
Lipofectamine alone	19.066 ± 0.665		
Untreated	19.266 ± 0.763		
PinX1-FAM-siRNA	17.166 ± 2.663**		

* vs untreated, *P *< 0.001; ** vs untreated, *P *> 0.05.

Apoptotic Index = apoptotic cell number/total cell number × 100%.

## Discussions

Telomerase is a special reverse transcriptase that is composed of RNA and protein and regulates the length of telomere. hTERT is the key component in telomerase and plays important role in genetic stability and maintainance of chromosomes. Studies have found that telomerase is almost not expressed in normal somatic cells, but its expression and activity are enhanced in most immortalized tumor cells [18,19]. Previous studies from our group and others have suggested that telomerase is closely related to the incidence of vast majority of human malignant tumors including nasopharyngeal carcinoma. Enhancement of its activity is the power source of constantly increased proliferation, invasion and metastasis of tumor cells. Therefore, downregulation of telomerase activity in tumor cells is one of the important therapeutic measures to inhibit tumor growth and has become a hot topic in tumor gene therapy. Our study and others have suggested that the targeted TK gene therapy under hTERT promoter or enhanced hTERT/CMV promoter can reduce telomerase activity, eventually leading to the death of tumor cells including NPC [6,7]. Thus, further exploration of specific telomerase inhibitors will be a new direction for future research.

LPTS/PinX1 is recently discovered in cell nucleus as a telomerase inhibitor that binds to Pin2/TRF1 complex *in vivo*. PinX1 gene is located on chromosome 8p22-23 region, which has high frequency of loss of heterozygosity (LOH) in a series of human cancer cells. LPTS is a novel liver-related putative tumor suppressor gene. The coding sequence of PinX1 is highly homologous to one of the LPTS transcripts, LPTS-L, and considered as a transcript of the same gene [20,21]. Some studies have found that PinX1 can attenuate telomerase activity, inhibit growth of tumor cells and induce apoptosis. Lack of endogenous PinX1 leads to increased telomerase activity and tumorigenicity in nude mice. Therefore, PinX1 is considered as telomerase inhibitor and tumor suppressor. Recent studies have also suggested that PinX1 as tubulin plays an important role in the maintenance of cell mitosis.

The mechanism of PinX1 functioning in tumor cells has not been fully elucidated. Some studies indicate that PinX1 gene can inhibit telomerase activity and induce cell apoptosis, and expression of PinX1 is negatively correlated with hTERT expression and telomerase activity in tumor cells. For examples, Liao et al. [10] reported that upregulation of LPTS-L by transfection of its expression vector in hepatoma cells can inhibit telomerase activity and induce apoptosis; Zhang et al. [22] reported that silencing PinX1 gene using short hairpin RNA can lead to significant shortening of telomere and growth inhibition of telomerase-positive tumor cell, but not telomerase-negative tumor cells, indicating PinX1 affects telomere length and tumorigenicity through regulating telomerase activity; Cai et al. [23] found that reduced PinX1 expression is highly correlated to the poor prognostic factors (such as lymph node metastasis and distant metastasis) in patients with ovarian cancer and considered as an independent factor for poor prognosis of patients with epithelial ovarian cancer; Wang et al. [24] constructed and transfected PinX1 and PinX1-siRNA eukaryotic expression vectors into gastric cancer cells and found that downregulation of PinX1 by transfection of PinX1-siRNA vector significantly enhanced telomerase activity compared with that of cells transfected with PinX1 vector, suggesting that PinX1 is a telomerase inhibitor and inhibits tumorigenesis and development possibly through telomerase/telomere pathway; Zhou et al. [25] believed that PinX1 inhibits telomerase activity by binding to hTERT through its TID domain, which consequently results in telomere shortening, cell senescence and increase of tumorigenicity in nude mice; Banik et al. [26] analyzed the relationship among PinX1, hTERT and hTR, and found that PinX1 can directly bind to hTERT and hTR, but the binding of PinX1 to hTR is dependent on the presence of hTERT. Inhibition of telomerase activity by PinX1 requires its binding to both hTERT and hTR. By contrast, some studies indicate that PinX1 expression is positively correlated to telomerase activity. For examples, Sun et al. [12] found that PinX1 mRNA level is closely related to hTERT mRNA level in differentiated acute promyelocytic leukemia cells and altered PinX1 expression is secondary response to changes of hTERT expression. In addition, some studies found that PinX1 is not the key factor in inhibition of telomerase activity and its function is rather related to gene polymorphism than to telomerase activity. For example, studies [14] on 159 cases of hereditary prostate cancer identified 39 polymorphisms during PinX1 sequencing; studies [15] on gastrointestinal cancer also found a missense mutation (AGC/TGC) out of 254 codons in 1 case of colon cancer and 1 case of esophageal cancer. The authors suggested that this mutation may be a benign polymorphism because neither de-hypermethylation on its promoter region nor 5-N-2-deoxycytidine treatment of a cell line affected PinX1 expression. In addition, Chang et al. [16] analyzed the function of PinX1 in medulloblastoma and found that 11 polymorphisms in its 7 exons and their splicing sites by direct sequencing and that telomerase activity was not inhibited and related to PinX1, indicating PinX1 did not play a key role in the process of medulloblastoma. Overall, the mechanisms of PinX1 on telomerase/telomere are complicated and may differ in different tumors. Recently, researches on PinX1 dynamics and function have also advanced our knowledges. Yuan et al. [17] have shown that PinX1 is located in the nucleolus and telomeres in the interphase and gathered around chromosome and outer plate of kinetochore in mitosis phase. Moreover, downregulation of PinX1 by siRNA leads to the aberrant separation of mitotic chromosome, suggesting that PinX1 play an important role in mitotic chromosome separation. Li et al. [27] further found that PinX1 is recruited to the area surrounding chromosome by nucleolin during mitosis to promote chromosomes congression. Chen et al. [28] found that PinX1 binds to Pin2/TRF1 and hTERT at different sites: LPTS/PinX1 (254- 289) binds to Pin2/TRF1, while LPTS/PinX1 (290-328) binds to hTERT. Binding of LPTS/PinX1 (290-328) to hTERT *in vitro *significantly inhibits telomerase activity, leading to telomere shortening and cell apoptosis.

In this study, we successfully constructed PinX1 expression vector pEGFP-C3-PinX1 and found its transfection into NPC cells significantly increased PinX1 mRNA level using RT-PCR, which laid the foundation for study on the roles of PinX1 in NPC cells. Our results also found that overexpression of PinX1 by transfection of pEGFP-C3-PinX1 into NPC cells significantly reduced hTERT mRNA level, telomerase activity, NPC cell growth, migration and wound healing ability, arressted NPC cells in G0/G1 phase and induced cell apoptosis, whereas those phenomena were not found in cells transfected with control vector pEGFP-C3 or treated with lipofectamine alone compared with nontreated NPC cells. These suggest that PinX1 is a potential inhibitor of telomerase activity, in consistence with many other previous reports on tumors.

To better understand the role of PinX1 on tumor cells, we also successfully established PinX1-specific siRNA PinX1-FAM-siRNA, transfection of which significantly silenced 70% of endogenous PinX1 mRNA in NPC cells (*p *< 0.001). However, PinX1 silencing did not alter telomerase activity, and NPC cell growth and migration. This discrepancy to previous studies [22,24] may be interpreted by the followings: 1) endogenous PinX1 level is already very low in tumor cells, therefore further downregulation has little effect on telomerase activity; 2) silencing PinX1 by transfection of PinX1-FAM-siRNA may be not long enough to observe substantial biological alterations of tumor cells. But no matter how, PinX1-FAM-siRNA can inhibit PinX1 expression *in vitro*. Although few studies have been conducted on PinX1 silencing, studies by Zhang et al. [22] and Wang et al. [24] confirmed PinX1 is closely related with telomerase activity.

## Conclusions

In conclusion, this study systematically analyzed the roles of PinX1 in NPC 5-8 F cells by oeverexpression or downregulation of PinX1 and found that PinX1 affects NPC cell growth, migration, wound healing ability and cell cycles by inhibiting telomerase activity, suggesting that PinX1 is a potential telomerase inhibitor and has potential therapeutic application in treatment of tumors including NPC. However, the study has some limitations: 1) PinX1 and it siRNA are only transiently transfected, their long-term effects on cells could not be observed; 2) the *in vitro *experiments may not reflect its role *in vivo*, therefore further evaluation of the experimental results is needed.

## Competing interests

The authors declare that they have no competing interests.

## Authors' contributions

XFL and CXS carried out the subtotal molecular genetic studies, participated in the design of the study, and performed the statistical analysis. ZW conceived of the study, and participated in its design and coordination and drafted the manuscript. YHQ carried out the cell culture. CSY participated in the PCR, MTT, telomerase activity and DNA sequence. JQW participated in study work in PinX1 With siRNA. PNZ carried out Transwell cell. HLW carried out PinX1 expression. All authors read and approved the final manuscript.
